# MicroRNA molecules as predictive biomarkers of adaptive responses to strength training and physical inactivity in haemodialysis patients

**DOI:** 10.1038/s41598-020-72542-1

**Published:** 2020-09-24

**Authors:** Ivana Spakova, Aurel Zelko, Miroslava Rabajdova, Peter Kolarcik, Jaroslav Rosenberger, Martina Zavacka, Maria Marekova, Andrea Madarasova Geckova, Jitse P. van Dijk, Sijmen A. Reijneveld

**Affiliations:** 1 Department of Medical and Clinical Biochemistry, Faculty of Medicine, Pavol Jozef Safarik University, Kosice, 040 11 Slovakia; 2 Department of Health Psychology and Research Methodology, Faculty of Medicine, Pavol Jozef Safarik University, Kosice, 040 11 Slovakia; 3 Graduate School Kosice Institute for Society and Health, Faculty of Medicine, Pavol Jozef Safarik University, Kosice, 040 11 Slovakia; 4 Olomouc University Society and Health Institute, Palacky University Olomouc, Olomouc, 771 11 Czech Republic; 5 2nd Department of Internal Medicine, Faculty of Medicine, Pavol Jozef Safarik University, Kosice, 040 11 Slovakia; 6 Fresenius Medical Care - Dialysis Services Kosice, Kosice, 040 11 Slovakia; 7 Department of Community and Occupational Medicine, University Medical Center Groningen, University of Groningen, Groningen, 9700 RB The Netherlands; 8 Clinic of Vascular Surgery, East Slovak Institute of Cardiovascular Diseases, Kosice, 040 11 Slovakia

**Keywords:** Molecular biology, Physiology, Health care, Nephrology

## Abstract

The miRNA-206 and miRNA-23a play an important role in muscle tissue hypertrophy, regeneration and atrophy. Both of these miRNAs have been highlighted as promising adaptation predictors; however, the available evidence on associations is inconclusive. Therefore, our aim was to assess the expression levels of these two miRNAs as predictors of change in muscle function during strength training and physical inactivity among dialysed patients. For this purpose, 46 haemodialysis patients were monitored for 12-weeks of either intradialytic strength training (EXG, n = 20) or physical inactivity during dialysis (CON, n = 26). In both groups of patients, we assessed the baseline expression levels of miRNA-23a and miRNA-206 and the isometric force generated during hip flexion (HF) contraction before and after the 12-week period. Among the EXG group, the expression of miRNA-206 predicted the change in HF (R^2^ = 0.63, *p* = 0.0005) much more strongly than the expression of miRNA-23a (R^2^ = 0.21, *p* = 0.027). Interestingly, baseline miRNA-23a (R^2^ = 0.30, *p* = 0.006) predicted the change in HF much more than miRNA-206 (*p* = ns) among the CON group. Our study indicates that the baseline expression of miRNA-206 could predict the response to strength training, while miRNA-23a could serve as a potential predictive marker of functional changes during physical inactivity in dialysis patients.

## Introduction

Patients In haemodialysis (CKD5-HD) patients, muscle functioning is strongly associated with hospitalizations^1^, mortality risks^1,2^, functional independence^3^, mobility and quality of life^4,5^. Chronic haemodialysis therapy is accompanied by a deterioration in muscle functions and weakness^6,7^. To counteract these health risks various interventional approaches have been proposed and strength training is considered to be an effective instrument for maintaining favourable muscle function in CKD5-HD patients^8^. Besides clinical efficiency in the prevention of functional loss, large individual differences in the physiological response to strength training and inactivity have been reported among CKD5-HD patients^9,10^.

Micro ribonucleic acids (miRNA) are synthesized, selectively packaged and actively secreted into circulation by a variety of cell types^11^. In circulation, miRNAs may be delivered to the recipient cells where they can regulate the translational activity of target genes^12^. Since their discovery, several miRNAs have been identified as biomarkers of normal development^13,14^ and pathophysiology of the muscular system^15–17^. More recent research has been conducted in the field of miRNAs functions in physiological adaptation during exercise interventions^18^, and miRNA-206 has been found to play an important role in myogenesis^19^, skeletal muscle regeneration^20^ and regulation of myostatin mRNA activity^21,22^. These physiological processes are necessary for the adaptation of muscle tissue after strength training. On the other hand, miRNA-23a interacts with the Muscle RING finger protein 1 (MuRF1) and the Muscle specific ligases atrophy F-box (MAFbx) signalling pathways, which closely regulate protein breakdown in skeletal muscle tissue^23^. Additionally, an miRNA-23a cluster affects proteolysis and mitochondrial integrity activity of inflammatory cytokines^24,25^, and miRNA-23a/miRNA-27a clusters have been considered for therapeutic use as an exercise mimetic for muscle wasting during critical and chronic disease conditions^26,27^.

**Figure 1:**
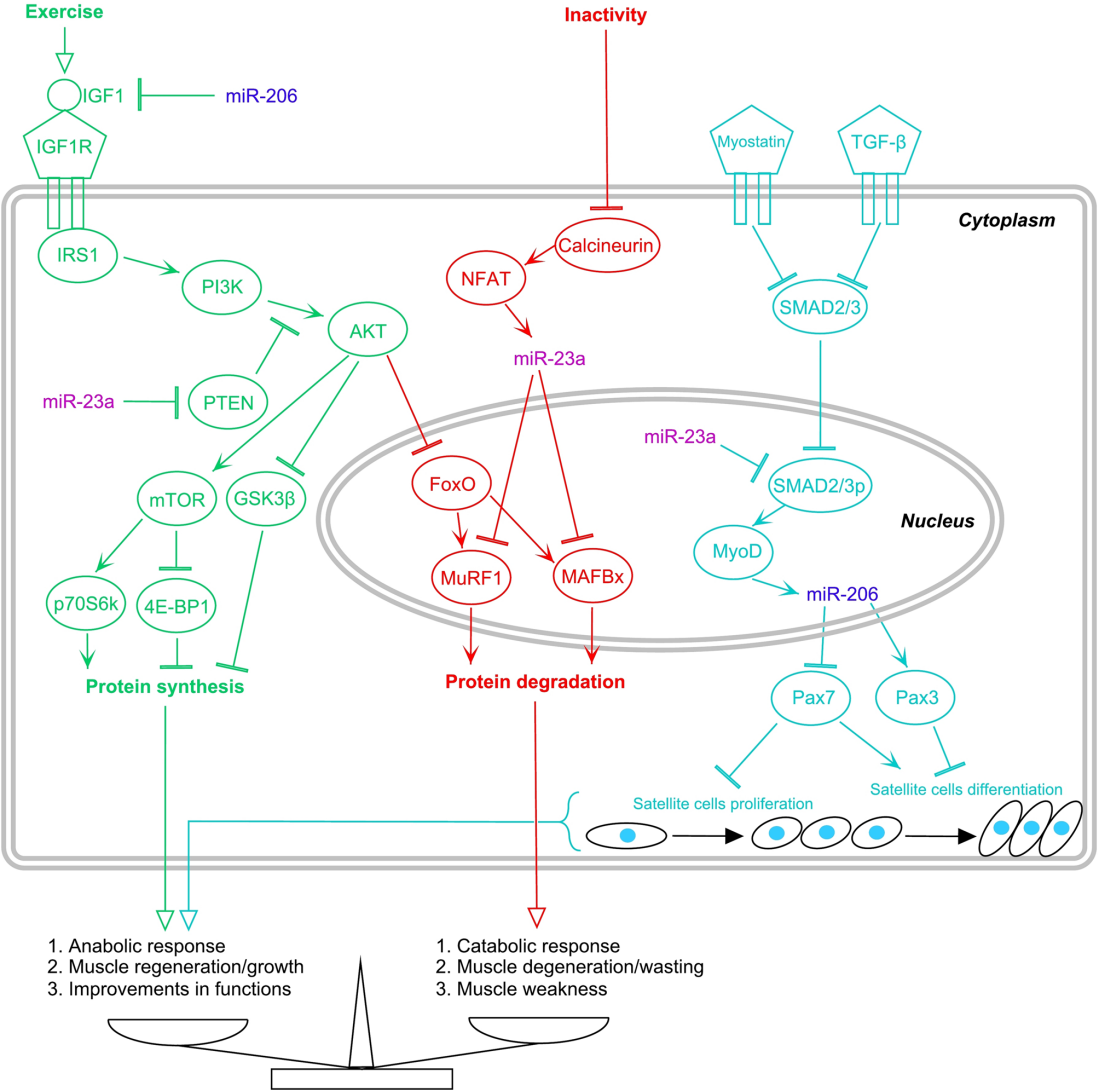
Summary of interactions and actions of miRNA-206 and miRNA-23a in the PI3K/Akt/mTOR, Calcineurin, Myostatin, and Transforming growth factor β signalling pathways of muscle cells (promotion →; inhibition  ).

Both miRNAs are physiologically active in muscle cells. They have the effect of the family member 2 and 3 (SMAD2/3) signalling pathways in cells (Fig. 1) through the promotion and inhibition of the Phosphatidylinositol-3-kinase/Phosphorylated serine-threonine kinase pathway (PI3K/Akt/mTOR) and Calcineurin.

The expression level of miRNA-206 has been correlated negatively with peripheral insulin sensitivity^28^ and reduced miRNA-206 levels increase insulin-like growth factor-1 (IGF-1) mRNA levels^29^ in the PI3K/Akt/mTOR signalling pathway. Myoblast determination proteins (MyoD) and the paired-box transcription factor (Pax) in the SMAD 2/3 signalling pathway are essential for muscle satellite cell proliferation and differentiation. Enhanced or reduced MyoD activity affects the expression of miRNA-206, and its altered expression causes resistance or acceleration of the apoptosis processes of myoblasts. The elevated expression of miRNA-206 suppresses the expression of Pax7, which restricts muscle satellite cells proliferation and promotes their differentiation^20,30,31^. A decrease in the expression of miRNA-206 increases the activation of Pax3, which suppresses the differentiation of muscle satellite cells^32,33^.

Activation of the Calcineurin (Cn) and the Nuclear factor of activated T cells (NFAT) signalling pathways increases miRNA-23a expression^34–36^. MuRF1 and MAFbx are muscle-specific ubiquitin ligases downstream from the Calcineurin signalling pathway and play critical roles during protein ubiquitination and muscle tissue atrophy^37^. MuRF1 and MAFbx are over-expressed during the activation of muscle atrophy, while the inhibition of MuRF1 and MAFbx attenuate atrophy processes^38,39^. Increased expression of miRNA-23a is able to inhibit the translational activation of MuRF1 and MAFbx and thereby suppress atrophy processes in muscle cells^32^. The increased concentration of miRNA-23a has been associated with down-regulation of SMAD2/3, with reduced concentrations of Phosphatase and tensin homolog (PTEN) and with the elevation of Akt concentrations^40^. Therefore, we hypothesized that the baseline expression levels of Homo sapiens-miRNA-206 and hsa-miRNA-23a may strongly predict the adaptation to strength training and physical inactivity in CKD5-HD patients.

Evidence is very limited on the predictive relationship between resting, pre-interventional, Homo sapiens (hsa-)miRNAs and the adaptive response to strength training or physical inactivity. Margolis et al. investigated and compared the predictive association between hsa-miRNAs and exercise-induced adaptations following acute strength training among young and elderly healthy subjects^41^. They found that dysregulation of hsa-miRNAs in the elderly served as predictive markers in age-associated declines in skeletal muscle mass, increased fat mass, and altered functional adaptability. However, only hsa-miRNA-206 was included in these predictive analyses. Therefore, in our study, we investigated the degree to which baseline levels of hsa-miRNA-206 and hsa-miRNA-23a modified adaptive response to strength training and to physical inactivity in CKD5-HD patients.

## Results

### Patient flow

For the present investigation, data were analysed on 20 experimental patients and 26 control patients, who completed all the assessments of outcomes and were identified as physically inactive in pre-intervention assessments.

### Patients’ baseline characteristics

At baseline, the mean age of patients in the study was 66.2 ± 9.4. Among the patients included for analysis, 54% were male and the BMI was 26.0 ± 5.8 kg/m^2^ (Table 1). Baseline relative expression levels of the studied hsa-miRNAs were comparable between the EXG and CON groups.

**Table 1:** Patients’ baseline characteristics and test of differences in these characteristics between the EXG and CON groups.

Variable	EXG (n = 20)	CON (n = 26)	*p value*
Age	63.9(9.9)	67.9 (8.9)	0.161
Gender (male/female)	11/9	14/12	0.938
Body mass index (kg/m^2^)	28.2 (6.5)	24.3 (4.6)	0.029*
Dialysis adequacy (Kt/V)	1.6 (0.4)	2.0 (0.3)	0.001^†^
Over-hydration index (%)	12.3 (6.2)	12.2 (6.9)	0.964
C-reactive protein (mg/l)	8.3 (10.7)	13.3 (14.4)	0.190
iPTH (pg/ml)	400.8 (375.3)	392.4 (460.4)	0.941
Albumin (g/l)	39.3 (3.0)	36.9 (4.4)	0.042*
Phosphates (mml/l)	1.6 (0.5)	1.5 (0.5)	0.396
Calcium (mmol/l)	2.1 (0.2)	2.3 (0.1)	0.001^†^
Hip flexion (N/kg)	107.6 (53.3)	96.3 (28.5)	0.397
hsa-miRNA-206 (log10)	42.7 (33.4)	17.5 (12.0)	0.013*
hsa-miR-23a (log10)	6557.2 (5139.0)	2132.8 (3944.0)	0.004^†^

Data are presented as mean ± standard deviation, hsa-miRNA relative expression data are presented as mean log10 (log10) of the respective miRNA ± standard deviation, *p* values determined by the unpaired Student´s t-test. iPTH, intact parathyroid hormone; EXG, experimental group; CON, control group. Differences between groups significant at *p* < 0.05 are marked by *. Differences between groups significant at *p* < 0.01 are marked by ^†^.

### Adaptive response during strength training intervention and physical inactivity

During the 12-week strength training intervention in EXG patients, the maximal force during isometric HF increased by 15.9 N (SD 36.7; pre: 107.6 N, post: 123.5 N; + 14.8%). In CON patients the 12-weeks of physical inactivity resulted in an increase of the maximal isometric force during HF by 3.0 N (SD 26.9; pre: 96.3 N, post: 99.3 N; + 3.1%). The relative force during isometric HF increased in the EXG group by 18.4 N/kg (SD 48.8; pre: 139.1 N/kg, post: 157.5 N/kg; + 13.2%) and decreased in the CON group by 1.5 N/kg (SD 40.3, pre: 141.4 N/kg; post: 139.8 N/kg; − 1.1%).

#### Do hsa-miRNAs modify changes in muscle function in all study subjects?

**Figure 2:**
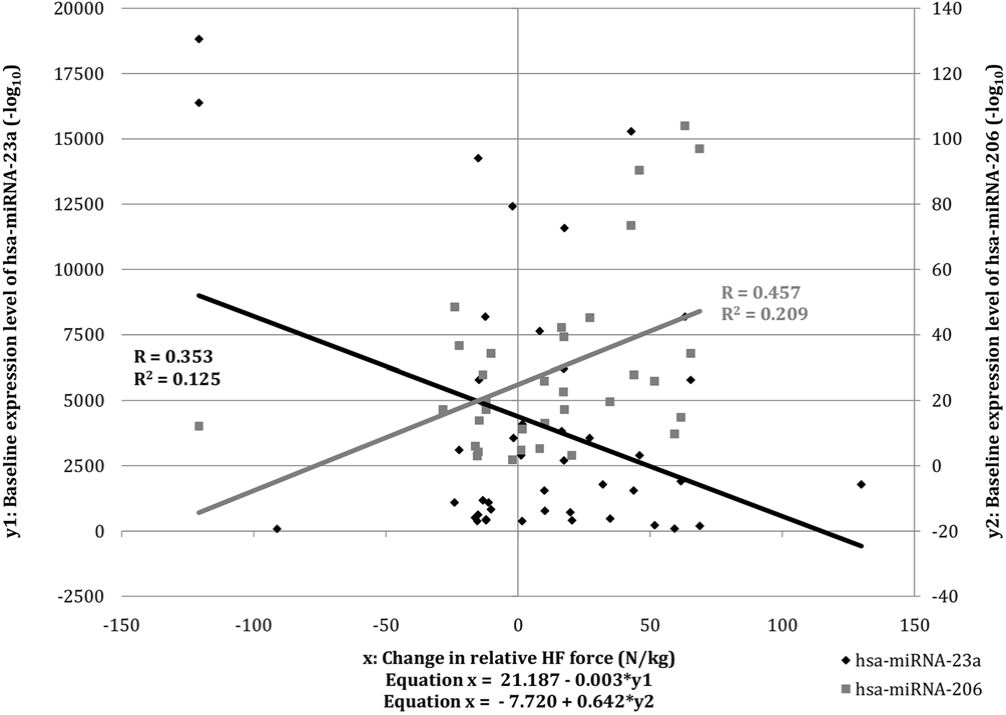
Regression analysis scatter plot of the relationship between the baseline expression level of hsa-miRNA-23a and hsa-miRNA-206 and the change in relative HF contraction force in all subjects.

Regardless of the patient’s allocation, the baseline expression levels of hsa-miRNA-206 and hsa-miRNA-23a modified a change of relative force during isometric HF contraction. They accounted for 31.2% of its variance (*p* = 0.003). Assessing the hsa-miRNAs separately, the baseline expression level of hsa-miRNA-206 explained 21.0% (*p* = 0.006) and the baseline expression level of hsa-miR-23a explained 12.5% (*p* = 0.020) of the variance in the change of relative force during isometric HF contraction (Fig. 2). Looking at the direction of associations with the change in patient’s muscle strength, we see that baseline expression of hsa-miRNA-206 was associated with an increase in isometric HF force and the baseline expression of hsa-miRNA-23a with a decrease in it.

#### Do hsa-miRNAs modify changes in muscle function during strength training?

**Figure 3:**
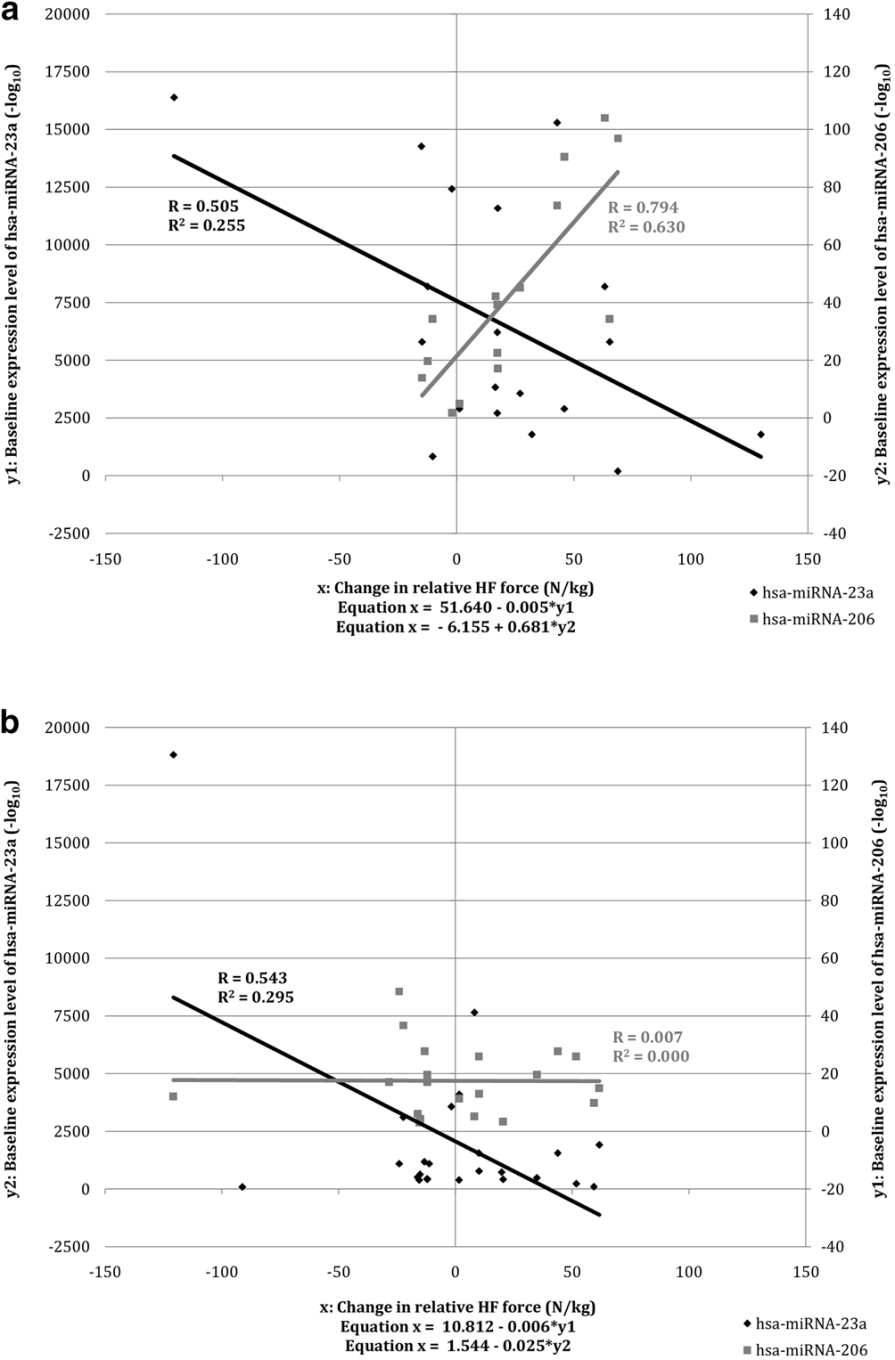
(**a**) Regression analysis scatter plot of the relationship between the baseline expression level of hsa-miRNA-23a and hsa-miRNA-206 and the change in relative HF force in the EXG group. (**b**) Regression analysis scatter plot of the relationship between the baseline expression level of hsa-miRNA-23a and hsa-miRNA-206 and the change in relative HF force in the CON group.

The baseline expression level of hsa-miRNA-206 (R^2^ = 0.63; *p* = 0.0005; Fig. 3a) modified the adaptive response during the strength training more strongly than the baseline expression level of hsa-miRNA-23a (R^2^ = 0.21; *p* = 0.027; Fig. 3a). Compared with data analysis in all subjects, the experimental group showed a stronger positive association between the baseline expression level of hsa-miRNA-206 and the change in relative force during isometric HF contraction, while the negative associations with the expression level of hsa-miRNA-23a remained moderate.

#### Do hsa-miRNAs modify changes in muscle function during physical inactivity?

The baseline expression level of hsa-miRNA-23a explained 29.5% of the variance in the change of relative force during isometric HF contraction (*p* = 0.006; Fig. 3b), while the baseline expression level of hsa-miRNA-206 showed no predictive association with the change in muscle functions (R^2^ = 0.00; *p* = 0.986; Fig. 3b). Comparing the control group with all subjects, the association between the baseline expression level of hsa-miRNA-206 and the relative change in HF contraction force disappeared, while the negative associations with the expression level of hsa-miRNA-23a remained moderate.

## Discussion

We performed a quasi-experimental study to access the relationship between the expression level of two hsa-miRNAs and functional adaptation during a period of physical activity and inactivity in CKD5-HD. We found that the baseline expression levels of hsa-miRNA-206 and hsa-miRNA-23a modifies changes in relative force during isometric HF contractions due to chronic resistance training and due to inactivity.

After the strength training intervention, the relative HF force of EXG patients increased considerably, by 18.4 N/kg. We found that these improvements in muscle function were strongly predicted by the baseline expression level of hsa-miRNA-206 among CKD5-HD patients. Previous research has shown that the hsa-miRNA-206 expression level predicts adaptation to strength training. That study compared the adaptability between young and elderly participants and reported that lower physiological responses to training and reduced anabolic signalling activity were accompanied by a lower baseline expression profile of hsa-miRNA-206^41^. This similarity with our results shows the independent role of hsa-miRNA-206 in physiological adaptation following physical activity intervention. The relationship between the hsa-miRNA-206 expression profile and the effects of physical activity may also be explained by in vitro studies and studies on non-human muscle cells^24^. In both models, the higher miRNA-206 expression levels were accompanied by lower insulin sensitivity and by lower IGF-1 mRNA levels^28,29^. Regular physical activity may improve insulin sensitivity and increase the expression of IGF-1 and thus make these patients much more adaptable to exercise interventions through higher activation of the PI3K/Akt/mTOR signalling pathway. Margolis et al.^41^ also observed a close physiological interaction between hsa-miRNA-206 and Akt activity. Moreover, the higher expression of hsa-miRNA-206 could inhibit Pax-7, which is known as a down-regulator of satellite cells proliferation in muscle cells^30,31^. The higher functional adaptation in patients with higher hsa-miRNA-206 may be explained by higher satellite cell proliferation activity via the TGF-β/SMAD2-3/MyoD signalling pathway. These physiological pathways indeed seem to be able to lead to modification of the responses to exercise.

We also found that after the period of physical inactivity, the relative HF force of CON patients slightly decreased, by 1.5 N/kg. We found that the deterioration in muscle function observed after the period of physical inactivity was predicted by the baseline expression level of hsa-miRNA-23a among CKD5-HD patients, i.e. that this modified the physiological response to inactivity. This may be explained by the modifications in gene expression and signalling pathways activity that were previously observed in patients during physical inactivity. For example, Duan et al.^57^ found that hsa-miRNA-23a was one of the differentially expressed genes associated with the loss of quadriceps muscle force during disease in patients during with an acute exacerbation of chronic obstructive pulmonary disease. A possible explanation for this may be that the higher expression of hsa-miRNA-23a down-regulated the activation of MuRF1 and MAFbx and decreased atrophy processes in muscle cells via the Calcineurin/NFAT signalling pathway^32^. It has been suggested that the higher expression of hsa-miRNA-23a may be positively associated with muscle functions. The observed negative associations between hsa-miRNA-23a and muscle function in our study are surprising and may be explained by the complementary effects of hsa-miRNA-23a in the PI3K/Akt/mTOR and the Myostatin/TGF-β/SMAD2-3/MyoD signalling pathways. In both pathways, higher expression levels of hsa-miRNA-23a down-regulates the anabolic and regenerative physiological effects via PTEN and SMAD2/3p. The decreased hsa-miRNA-23a expression in inactive patients may up-regulate both signalling pathways and help muscle cells to maintain sufficient anabolic signalling activity for the preservation of muscle cell volume and functions during physical inactivity.

This is the first study which examines associations between the expression profile of hsa-miRNA-206 and hsa-miRNA-23a and changes in muscle function during exercise intervention and inactivity in dialysed patients. We found that baseline expression of hsa-miRNA-206 is a strong predictor of functional adaptation during strength training and that hsa-miRNA-23a is a moderate predictor of functional changes during physical inactivity.

To the best of our knowledge, this study is the first to identify the modifying effects of the baseline expression levels of hsa-miRNA-206 and hsa-miRNA-23a on the change of lower extremity muscle strength during physical activity and inactivity among CKD5-HD patients. The sample size and closely monitored interventional conditions are major main strengths of this study. Given that our findings are based on a limited number of miRNAs and no encoded proteins analysis, the results from our data analyses should be treated with considerable caution.

Our findings of associations between muscle function changes and hsa-miRNA-206 during strength training and hsa-miRNA-23 during physical inactivity could be used in the development of molecular biology instruments to effectively slow down and prevent the muscle function deterioration that frequently accompanied acute life-threatening and chronic disease conditions. Our findings are also important for the management and implementation of an intradialytic exercise intervention on dialysed patients. In patients with a higher baseline expression of hsa-miRNA-206, the application of strength training will likely yield favourable physiological effects and should be strongly recommended. In patients with low baseline expression of hsa-miRNA-206, the training intervention could be modified (length of intervention, type of exercise, dosage of exercise, etc.) to achieve similar adaptation effects compared to the high-expression patients.

## Methods

### Study design

We embedded the assessment of the hsa-miRNA predictive value in a quasi-experimental, two-group, pre-post comparative study at three dialysis centres in Slovakia (Fresenius Medical Care Dialysis Services in Kosice, Logman East in Kosice and Fresenius Medical Care Dialysis Services in Banska Bystrica). The study design and protocol were reviewed and approved by the Ethics Committee of Pavol Jozef Safarik University in Kosice (approval no. 14N/2017) and registered at ClinicalTrials.gov (ID:NCT03511924). All methods, assessments and data acquisitions were conducted according to the relevant ethics guidelines and regulations, based on the Declaration of Helsinki (1975, as revised in 2013) and followed the study protocol^42^.

### Participants

We included patients with end-stage renal disease who were over 30 years of age and had been receiving treatment by maintenance dialysis therapy for at least the last three months. Potential participants were screened and selected through their nephrologists. Individuals were excluded if they had lower extremity amputation, severe dementia or retardation, an acute intercurrent disease or if their probability of one-year mortality was higher than 25% according to the Charlson Comorbidity Index^43^. All assessments and training sessions were done at the three cooperating dialysis centres. All 198 CKD5-HD patients were screened and selected according to the inclusion and exclusion criteria at these centres, yielding 126 eligible patients (63.6% eligible patients). These received oral and written information about the possibility of participating in the study, leading to 90 patients signing a written informed consent (71.4% response rate) prior to the study.

### Patient allocation

Patients attending dialysis therapy at both sites in Kosice were allocated to the experimental group (EXG, n = 57), while patients from the Banska Bystrica dialysis centre were allocated to the control group (CON, n = 33). After the allocation procedure, the investigatory team members and participating patients were informed about the group assignment structure^58^.

### Intervention

The intervention has been described in details in study protocol publication^42^. Patients allocated to the EXG participated in a 12-week intradialytic resistance training (IRT) programme, which they performed under the supervision of training assistants, three times per week during the dialysis therapy. IRT sessions lasted 40 min and were composed of 3-min of warming-up, 30-min of conditioning and 7-min of cooling-down and stretching. To perform effective exercises in a supine position during dialysis, we used external pressure generated by elastic bands and over-balls (TheraBand, OH, USA). These external loading resources (bands and balls) were fixed on the construction of the dialysis bed and during exercises, patients pulled or pushed against them. The applied intensity of IRT was medium corresponding with 11–14 out of 20 at the rate of The Borg Scale of Perceived Exertion^44^. The progress of the IRT programme strongly depended on the patient’s physical abilities and clinical condition. During the first two weeks of the IRT programme, patients performed per session three sets (12–15 repetitions each) of three different exercises for the lower extremity muscles (I. unilateral push and pull of the over-ball against a leg board, II. bilateral knee squeeze of the over-ball, and III. unilateral straight leg raise against the band pressure). Once a patient was capable of safely completing the planned programme for the session concerned, the numbers of repetitions in the next session increased with three repetitions for each exercise. If a patient reached the maximum number of repetitions per exercise (18 repetitions) during a session, then for the next session the number of sets was increased by one set, and the initial number of repetitions per exercise became 12. When the patient was able to perform five sets with 18 repetitions for each exercise, we made the IRT harder by applying a stiffer elastic band or an over-ball with higher hardness. If a patient failed to complete the entire training session, or had obvious difficulties, the IRT was facilitated by lowering the number of repetitions per set, or of sets, or by the application of softer elastic bands and over-balls^42^. This methodology of training progressivity enabled us to maintain the patient’s safety during IRT and ensured the intensity of training to be between “moderate” and “hard”.

### Control conditions

Patients allocated to the CON group received their standard nephrology care and remained physically inactive during dialysis. Through the 12-week control period, all CON patients maintained their standard treatment regimen and their customary dietary and physical activity patterns. The CON patients were informed about the clinical benefits and effects of regular physical activity in CKD5-HD patients, and during the control period they were received increased attention from the research team members^58^.

### Measures

The primary outcome of the study was the relative change in maximal isometric force generated during hip flexion (HF) contraction. The maximal isometric forces (1RM) generated during HF of patients were assessed by a hand-held dynamometer (Universal digital force gauge HF 500, SAUTER GmbH, Balingen, Germany). During the assessments, patients were in a supine position and held the dominant leg in a straightened position, while the dynamometer was placed proximally to the ankle, on the anterior surface of the lower leg. The patients were instructed to perform a maximal isometric contraction and hold it for 5 s. The tests were repeated with 30-s rest intervals, and the higher values of two consecutive tests were used for the analysis as absolute values of 1RM (measure unit: Newton; N). The absolute values of 1RM were subsequently divided by the subject’s body weight to determine relative 1RM forces (Newton per kilogram; N/kg). Changes in the relative 1RM forces during HF were calculated for experimental and control conditions as the post-intervention measure minus the baseline measure.

The potential modifiers of the study included the resting, pre-interventional expression profile of hsa-miRNA-206 and hsa-miRNA-23a. Immediately after venous blood collection by a closed sampling system at the dialysis centres, blood was distributed into the dipotassium ethylenediaminetetraacetic acid anti-coagulation containing tubes and into tubes with a pro-coagulation solution. Samples were centrifuged at 3500×*g* at 4 °C for 3 min and the extracted samples containing separated plasma and serum were immediately stored at − 80 °C. All plasma samples were processed and analysed in a single laboratory batch 12 months after the first sample collection. During processing in the lab, samples were defrosted on ice and then 220 ml aliquots from the samples were centrifuged at 4 °C for 10 min (1000×*g*) to remove of potential residues. In the next step, total RNA including target miRNAs was isolated from 200 ml of plasma with the miRNeasy Serum/Plasma Kit (Qiagen, Hilden, Germany). The miRNA isolation efficiency was measured using the Qubit microRNA assay kit (Thermo Fisher Scientific, MA, USA).

Isolated RNA samples were processed for reverse transcription of hsa-miRNA-23a and hsa-miRNA-206 into cDNA using the TaqMan reverse transcription reagents kit (Thermo Fisher Scientific, MA, USA) and miRNA-specific primers (Thermo Fisher Scientific, MA, USA). The Reverse PCR was performed in a thermocycler in a three steps reaction as follows: Primer Extension 16 °C for 30 min, cDNA synthesis 42 °C for 30 min, Reaction Termination 85 °C for 5 min, and cooling to 4 °C. The concentrations of the target miRNAs were analysed by using quantitative real-time PCR by application of TaqMan Master Mix II not containing uracil-N-glycoslyase and specific TaqMan MicroRNA Assays on a Rotor-Gene Quantitative-Polymerase Chain Reaction Thermocycler (Qiagen, Hilden, Germany). The RT-PCR analyser was set in four steps as follows: Hold 1 (50 °C, 2 min), Hold 2 (95 °C, 10 min), 40 Cycles (95 °C, 15 s, 60 °C, 1 min), and Hold 3 (40 °C, 10 min).The analyses of the hsa-miRNAs concentrations were performed twice for each sample, with the acceptable intra-assay variation set at 4%. Final quantification of individual hsa-miRNAs levels was done using the Rotor-Gene Q software (Qiagen, Hilden, Germany). The comparative threshold cycle (CT) method, with “housekeeping” references RNU44, U6, ^ΔCT−40^ and the average ΔCT of analysed samples as the endogenous control were used for individual hsa-miRNAs quantification^46–47^. After this normalization, the delta threshold cycle (ΔCT) values were used to determine the delta delta threshold cycle (ΔΔCT) and obtain the relative amount of the miRNA to be determined using the formula for relative quantification (target gene 1) = 2^−ΔΔCT(target gene 1)^^48,49^.

Background variables regarded (a) patient’s age and gender, (b) body composition parameters (body weight and body height) and (c) nephrological clinical data containing over-hydration status, dialysis adequacy (Kt/V) and concentrations of C-reactive protein, parathyroid hormone, albumin, phosphates and calcium. These were extracted from patients’ electronic medical record right before the start of the intervention. The body mass index was calculated as body weight in kilograms divided by the square of the body height in metres (BMI, kg/m^2^). Background variables regarding the inactivity prevalence were assessed during an investigator-patient interview. Individual physical activity reports referencing the frequency, duration and type of physical activities were constructed following the instructions of the Global Physical Activity Questionnaire^50^. A patient was considered to be physically inactive if he or she reported less than 3 × 30 min of moderate-intensity physical activity per week^51,52^.

The primary outcome measures were collected in both groups before and after the experimental and control conditions. The modifiers and background variables were collected only before the start of the experimental or control conditions.

### Sample size calculation

The study sample size was determined based on the results from previously published articles, which reported changes in lower extremity muscle strength in CKD5-HD patients who underwent intradialytic exercise intervention^53–55^. We estimated that to have 80% power to detect an effect size of 0.60 in a change of a primary outcome between EXG and CON (two-tailed, α level of 0.05) at *p* < 0.05, 27 patients were required in each group. Anticipating a 70% retention rate during the study, we planned to enroll at least 39 patients in each group.

### Statistical analysis

First, we assessed background variables and compared those of the two study groups for possible differences using χ^2^ tests for categorical (binary) variables and the Student´s t-test for continuous variables. Second, we assessed whether baseline expression profiles of hsa-miRNA-206 and hsa-miRNA-23a modified the relative change in isometric HF force, overall and for the experimental and control conditions separately. We did so by using linear regression analyses of the change in the isometric HF by group condition (interventional vs. control condition), and whether baseline expression profiles of hsa-miRNA-206 and hsa-miRNA-23a modified this association. The level of significance was set at an α level of 0.05. Data analyses were carried out using the statistical software package IBM SPSS 22.0^56^.

## Data Availability

The datasets generated during and/or analysed during the current study are available in the Zenodo repository (https://doi.org/10.5281/zenodo.3678285).
